# Research advances of copper homeostasis and cuproptosis in orthopedic diseases

**DOI:** 10.1038/s41420-025-02921-y

**Published:** 2026-05-19

**Authors:** Jiwei Huang, Wenrui Zhang, Weitong Gao, Tao Guo, Zhengcai Han, Yan Yu, Haiyan Zhao

**Affiliations:** 1 The First Clinical College of Medicine, Lanzhou University, Lanzhou, China; 2 Department of Orthopedics, The First Hospital of Lanzhou University, Lanzhou, China; 3 Department of Medical Oncology, Harbin Medical University Cancer Hospital, Harbin, China

**Keywords:** Cell death, Diseases

## Abstract

Copper is an essential trace element that plays a significant role in human physiological functions. However, abnormal accumulation of copper can trigger a programmed cell death mechanism known as cuproptosis, which has a significant pathological impact, especially in orthopedic diseases such as osteoarthritis (OA), rheumatoid arthritis (RA), osteosarcoma (OS), and osteoporosis (OP). Cuproptosis not only causes damage to cell membranes, proteins and DNA through oxidative stress mechanisms, but also triggers apoptosis by disrupting mitochondrial function, thereby further exacerbating the structural damage of bone and cartilage tissues. The cuproptosis-related genes (CRGs) exhibit unique biological functions in regulating these pathological processes, and thus become potential diagnostic markers and therapeutic targets. In this regard, we have proposed an innovative therapeutic approach that utilizes the water gel sustained-release technology to load natural antioxidants. The aim is to achieve multi-dimensional targeted cuproptosis through improving drug delivery. Furthermore, natural substances such as sage extract, cinnamon and probiotics have demonstrated the potential for potent antioxidant and anti-inflammatory effects, providing a foundation for future therapeutic strategies. A key challenge for these strategies will be to precisely modulate copper levels to induce therapeutic cuproptosis in pathological cells (e.g., in osteosarcoma or hyperactive osteoclasts) while preserving the essential physiological functions of copper in normal bone and cartilage remodeling. Exosomal miRNAs provide a new perspective for intervening in the pathologies caused by copper through regulating copper homeostasis and related signaling pathways. Future research will focus on optimizing the combination application of these natural drugs, exploring the potential of different copper chelators and pioneering the design of innovative biomaterials, in order to achieve better control of cuproptosis and innovative progress in the treatment of orthopedic diseases.

## Introduction

Copper is an important element in the human body, existing in various tissues. It is mainly located in the interstitial spaces between cells and plasma. The physiologically relevant oxidation states are Cu2+ and Cu+, among which Cu+ is the predominant form in the cytoplasm. Copper, as a necessary cofactor, regulates the functions of enzymes and plays a significant role in various cellular processes such as mitochondrial respiration, antioxidant defense, signal transduction, kinase activity, autophagy and protein quality control [1]. Both deficiency and excess of copper can lead to imbalance of internal homeostasis and are associated with diseases such as oxidative stress and cytotoxicity [2, 3]. Under normal circumstances, the intracellular copper ion concentration is relatively low. An excessively high level of copper ions may lead to cytotoxicity and even cell death. In 2022, Peter Tsvetkov and his team discovered a new form of programmed cell death, which they named cuproptosis, due to the excessive copper levels. The intracellular copper promotes the thioctic acidylation aggregation process of mitochondrial-related proteins, thereby enhancing the decomposition of Fe-S cluster proteins and the protein toxicity response, ultimately leading to cell death. Therefore, it is called cuproptosis [4]. Cuproptosis not only increases the generation of reactive oxygen species (ROS), causing damage to cell membranes, DNA and proteins, but also induces apoptosis by disrupting mitochondrial energy metabolism and amplifying inflammatory responses. These pathways have been confirmed to persist continuously in osteoporosis, osteoarthritis and rheumatoid arthritis and other orthopedic diseases, posing a serious threat to the physiological structure of bones and cartilage. Although metal ions have been widely applied in cartilage repair, the research on metal ion therapy in vivo is still scarce. The existing studies are mainly confined to in vitro cell research. Studies have shown that the imbalance of copper ion levels may affect the process of bone formation. The copper homeostasis imbalance and copper death can affect osteoblasts, osteoclasts and chondrocytes, thereby influencing the development of bones and cartilage. The current therapeutic strategies often focus on the known copper homeostasis imbalance and the pathological effects caused by copper death, but the clinical outcomes are rather limited. Research indicates that further exploration of CRGs (cuproptosis genes) in relation to the pathological mechanisms of orthopedic diseases and related therapeutic targets, as well as innovative applications of new biomaterials and natural drugs, may provide new possibilities for personalized medicine. This article reviews the latest progress of copper homeostasis and cuproptosis in orthopedic diseases, explores potential therapeutic strategies and future research directions, aiming to achieve effective management of cuproptosis and the related orthopedic pathologies through multi-level and multi-dimensional intervention measures. Future research will focus on optimizing the combination application of these natural drugs, exploring the potential of different copper chelators and pioneering the design of innovative biomaterials, in order to achieve better control of cuproptosis and innovative progress in the treatment of orthopedic diseases.

Although studies have confirmed that imbalances in copper ion levels affect the process of bone formation, a deeper question remains: why do different types of bone cells exhibit varying sensitivities to disruptions in copper homeostasis? We speculate that this may be related to the unique metabolic characteristics and expression profiles of copper-related genes (CRGs) in each cell type. For example, osteoprogenitor cells with high metabolic activity may be more susceptible to copper-induced cell death due to their reliance on mitochondrial respiration, whereas relatively quiescent mature bone cells might have greater tolerance to such disturbances. This cell type-specific response could be key to understanding why copper imbalance can simultaneously lead to different pathological phenotypes such as osteoporosis and osteoarthritis.

## Copper metabolism and copper homeostasis

Under normal circumstances, Cu maintains a stable state within the cells. The stability of Cu mainly depends on three copper transporters, namely SLC31A1, ATP7A/B and ATP7B. SLC31A1 is responsible for copper intake, while ATP7A/B is responsible for copper efflux. The Cu ion carrier escholimol can transport Cu2+ into cells, resulting in the accumulation of Cu within the cells. However, enelastatin (BSO) indirectly affects the coordination between GSH and intracellular Cu ions by inhibiting the synthesis of glutathione (GSH). In the case of copper homeostasis imbalance, on one hand, when Cu2+ accumulates excessively in cells that rely on mitochondrial respiration, Cu2+ binds to S-acylated dihydroacylamine S-acetyltransferase (DLAT), inducing abnormal oligomerization of DLAT. The rise in insoluble DLAT results in cytotoxic effects and triggers cell mortality. On the other hand, ferredoxin 1 (FDX1) reduces Cu2+ to more toxic Cu+, thereby inhibiting the synthesis of Fe-S cluster proteins and inducing cell death. Furthermore, a relationship exists between CRGs and cytokines that impact synovial cells, which in turn can affect multiple cell types within the joint, including macrophages, monocytes, and neutrophils.

## Disorder of copper in bone and cartilage

Around two-thirds of the copper in the human body can be found in the muscles and skeleton. Numerous studies have confirmed that copper plays a significant role in regulating bone metabolism, influencing collagen synthesis and matrix metabolism, and supporting bone density and development [5, 6]. Copper is also crucial for the balance of the cartilage structure, through the assistance of lysyl oxidase (LOX) in cross-linking collagen [7]. The copper homeostasis is mainly dependent on the transporters SLC31A1 and ATP7A/B. However, disruption of copper homeostasis, such as excessive accumulation or deficiency of copper, can lead to copper concentrations within cells exceeding or falling below the threshold maintained by the homeostatic mechanism. This occurrence may result in harmful impacts on bone and cartilage cells [8]. The research has found that the copper concentration in articular cartilage is higher than that in other regions. Cu2+ is beneficial for cartilage regeneration and tissue interface repair, and enhances the chondrogenic differentiation of bone marrow stem cells [9–11]. The deficiency of copper affects copper-dependent enzymatic reactions, such as LOX, which damages and weakens the bone matrix, and makes cartilage brittle and prone to damage in integrity [9–12]. High copper levels inhibit the transformation of rat bone marrow mesenchymal stem cells into osteoblasts. This effect is achieved by influencing the stability of HIF-1α and Runx2 and reducing osteoclasts [13, 14]. Copper protein participates in the clearance of ROS, maintaining mitochondrial function and cellular metabolism, but the conclusions vary [15]. The deficiency of copper leads to an imbalance in redox reactions, affecting osteoblastogenesis and collagen stability, and weakening bone strength [16–19].

In tissue engineering, metal ions enhance cell functions, but the research on copper is rather limited. Whether increasing copper in defective cartilage promotes recovery is worthy of study. Although copper overload can damage cartilage, especially in patients with WD or OA, the relationship between copper levels and injury has been observed [20–23]. The introduction of copper into chondrocyte cultures alters the cytoskeleton. The effect and the specific mechanism remain to be explored. Through innovative research on related scaffolds, new insights are expected to be gained [24, 25]. Nevertheless, the precise manner in which copper deposition influences bone and cartilage is yet to be explored. Whether to conduct in-depth research on the impact of cell therapy on copper levels, whether in the system itself, or in terms of related scaffolds, alternative materials and similar breakthrough innovations, in anticipation, these efforts will bring new insights to the upcoming research work.

## The mechanism of cuproptosis in the pathogenesis of orthopedic diseases

Copper, an essential trace element, is crucial for the physiological functions within living organisms. However, the pathological mechanism of cuproptosis caused by abnormal copper accumulation has become one of the notable pathological mechanisms in orthopedic diseases. Copper catalyzes the peroxidation reaction to generate reactive oxygen species (ROS), thereby triggering severe oxidative stress. This kind of oxidative stress can damage cell membranes, proteins and DNA, affecting the functional stability of osteoblasts and chondrocytes.

Consequently, it plays a key pathogenic role in various orthopedic diseases such as osteoporosis, osteoarthritis and rheumatoid arthritis. Furthermore, cuproptosis also leads to the disruption of mitochondrial function, resulting in metabolic disorders of energy metabolism and triggering apoptosis of cells. As a consequence, it weakens the structural integrity of bones and joints. In the cellular signaling pathways, copper amplifies the inflammatory response and cell apoptosis pathways by activating key factors such as NF-κB and HIF-1α, thereby further aggravating the disease progression. These mechanisms work together to cause complex pathological effects of osteonecrosis in bone and cartilage tissues, providing a new perspective for the formulation of clinical intervention strategies.

### Cuprotosis in bone and cartilage biology

**Fig. 1:**
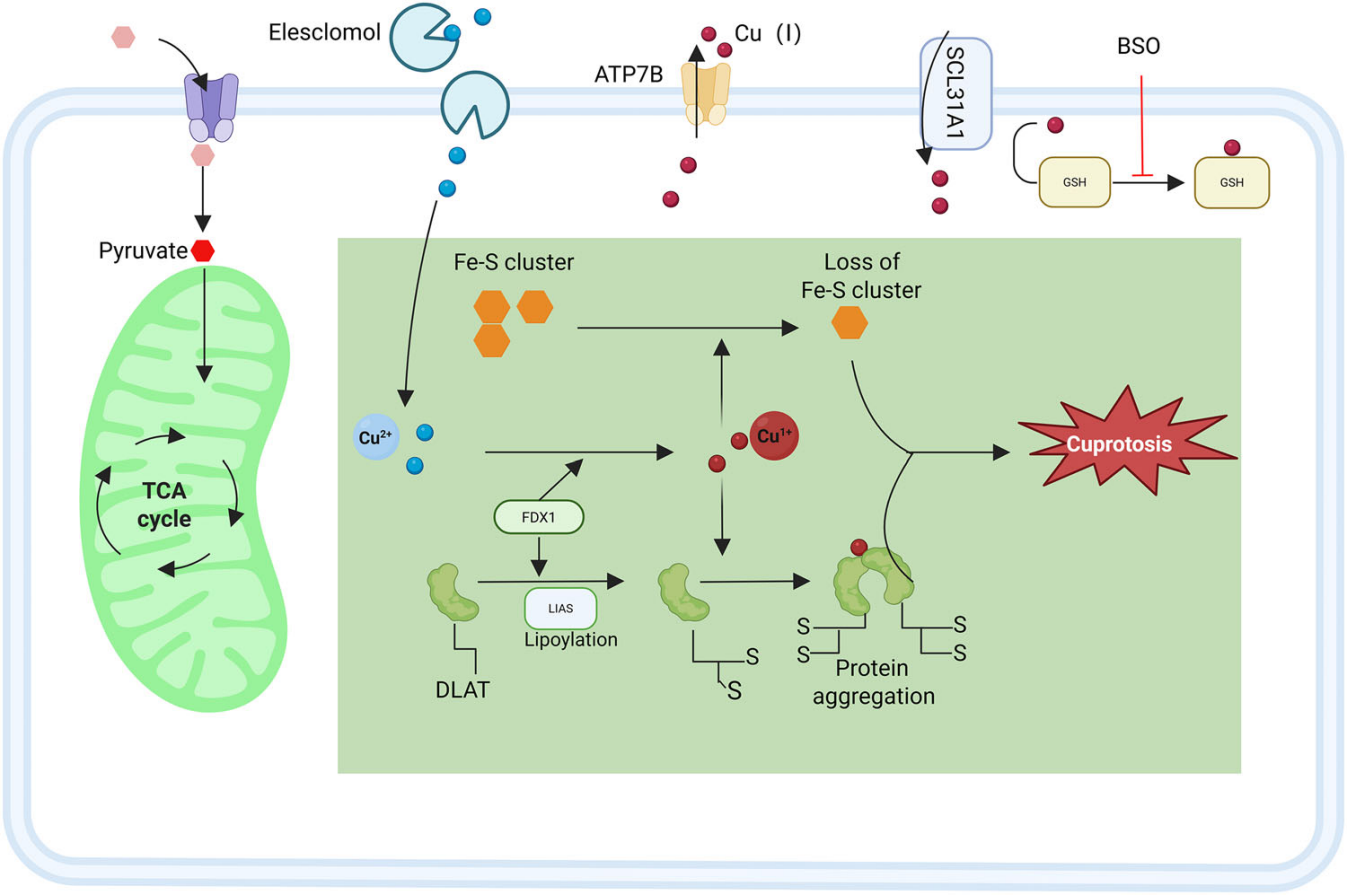
The mechanism of cuproptosis and its impact on OA. DLAT Dihydroceramide S-acetyltransferase, FDX1 Ferroxidase 1, LIAS Thiosulfate synthase, TCA Tricarboxylic acid cycle, Fe-S cluter Iron-sulfur cluster protein. Under ordinary conditions, copper maintains a stable level inside cells, The steadystate of Cu predominantly relies on three copper transporters, namely ATP7A/BandSLC31A1. SLC31A1 is accountable for the uptake of copper into cells, whereas ATP7A/B is in charge of copper efflux. The Cu ion carrier ethylchelmonate is capableof transporting Cu2+ into the cell, resulting in the accumulation of Cu within the cell. BSO restrains the chelating activity of the intracellular Cu chelator GSH. In the event of disruption of copper equilibrium, on the one hand, when there is an excessiveaccumulation of Cu2+ in cells that are dependent on mitochondrial respiration, Cu2+combines with sulfinic acid DLAT, resulting in abnormal oligomerization of DLAT. The augmentation of insoluble DLAT gives rise to cytotoxicity and triggers cell death; On the other hand, FDX1 converts Cu2+ into a more toxic form of Cu+, this will result in the suppression of Fe-S cluster protein synthesis and trigger cell death.

Cuprotosis is a unique form of cell death triggered by copper accumulation, which was first proposed by Tsvetkov et al. in 2022. Copper enters cells through Cu2+ carriers, causing a decrease in GSH and an increase in Cu2+ levels. This affects the binding of α-lipoic acid in the tricarboxylic acid cycle, leading to protein aggregation and a reduction in Fe-S cluster proteins, thereby triggering protein toxicity stress and ultimately causing cell death [4] (Fig. 1). In the past few years, the function of copper in the biology of bones and cartilage has begun to be uncovered. CRGs are cuprotosis-related genes, such as DLAT, MTF1, GLS, etc., which affect the functions of osteoblasts, osteoclasts and chondrocytes. High expression of DLAT leads to cartilage damage, while its inhibition can reduce cell death [26]. MTF1 is expressed in chondrocytes and affects osteophyte formation and cartilage injury [27]. GLS and glutamine metabolism determine cell fate and regulate osteogenic differentiation of osteoblast precursors [28, 29]. CDKN2A encodes the P16 (INK4A) protein, which affects osteoblast mineralization and senescence [30]. PDHA1, PDHB, DLD and other genes are involved in regulating the differentiation of osteoblasts and osteoclasts as well as the release of inflammatory factors [31]. In the synovial tissue, some CRGs such as FDX1 and LIPT1 are highly expressed, which affects the function of the synovium [32, 33]. Currently, the role of CRGs in bone and cartilage biology is not fully understood, and the underlying mechanism of CRGs remains to be elucidated. Nevertheless, previous studies have shown that many genes have direct or indirect effects on bone formation and bone resorption. In the future, these related genes will have significant prospects for the advancement in the fields of bone and cartilage biology. Therefore, additional research investigation is required.

Current understanding of the role of CRGs in osteochondral biology is still in its early stages, but exploration of their mechanisms has shown great potential. Notably, different CRGs may form synergistic regulatory networks: for example, copper toxicity mediated by FDX1 might intersect with glutamine metabolism regulated by GLS, jointly determining the differentiation fate of osteoblasts. The interactions within this gene network are far more complex than a simple sum of individual gene functions. Future research needs to move beyond traditional single-gene analysis approaches and adopt systems biology methods to unravel the regulatory networks of CRGs, which will provide new perspectives for understanding copper homeostasis regulation in the skeletal system.

### Copper-mediated oxidative stress

Copper, as a strong oxidant, can catalyze peroxidation reactions to generate reactive oxygen species (ROS), which can damage cell membranes, proteins and DNA. Oxidative stress is regarded as one of the important pathogenic factors contributing to osteoporosis. It causes bone resorption by disrupting the stability of osteoblasts and bone matrix, thereby leading to a decrease in bone density [34]. In patients with osteoarthritis (OA), abnormal accumulation of copper leads to oxidative stress, which is regarded as one of the key factors in the pathological process of OA. Copper directly damages cells by enhancing the generation of ROS, especially leading to impaired function of chondrocytes and increased apoptosis [35]. For patients with rheumatoid arthritis (RA), excessive copper intake can also catalyze peroxidation reactions and generate excessive oxidative stress. This can stimulate the production of more pro-inflammatory factors such as TNF-α and IL-1β by synovial cells and infiltrating immune cells, thereby exacerbating synovial inflammation [36, 37]. The research also indicates that copper-induced cell death triggered by oxidative stress is one of the key factors in the process of femoral head necrosis [38]. The oxidative stress exacerbated by ROS generation in copper not only damages the DNA and proteins of osteocytes, but also affects the metabolic activities of osteoblasts and chondrocytes, leading to the death of osteocytes [39]. In bone tumors, excessive copper can attack the DNA, proteins and cell membranes of tumor cells by enhancing oxidative stress. This oxidative stress not only affects tumor cells but also acts on normal cells in the tumor microenvironment [40, 41].

### Mitochondrial dysfunction

Cuproptosis is a kind of programmed cell death mechanism that leads to cellular dysfunction by destroying mitochondria. The core mechanism is that copper ions bind to the lipidated proteins inside mitochondria, causing abnormal aggregation of proteins and thereby disrupting the mitochondrial membrane potential. This functional disorder will disrupt the cellular energy metabolism, leading to an increase in osteoblast apoptosis and a reduction in energy supply, thereby weakening the stability of the bone structure [42]. During the process of osteoarthritis (OA), mitochondrial metabolic disorders directly affect the survival and metabolic functions of chondrocytes, thereby exacerbating the progression of the disease [43]. Furthermore, mitochondrial damage not only disrupts the function of the respiratory chain in cells, resulting in insufficient ATP production, but also aggravates the damage to the synovial membranes of joints, indirectly promoting the degeneration of joints related to rheumatoid arthritis (RA) [44]. In avascular necrosis of the femoral head (AVNFH), this energy metabolism disorder leads to insufficient energy supply for osteocytes, resulting in increased apoptosis and aggravating the disease condition [45]. In the context of tumors, the binding of copper to mitochondrial proteins leads to the reprogramming of tumor cell metabolism, thereby affecting their proliferation and growth [46]. Overall, cuproptosis plays a key pathogenic role in various diseases by inducing mitochondrial dysfunction and energy metabolism imbalance.

### Regulation of cellular signaling pathways and inflammatory responses

The accumulation of copper ions triggers a series of inflammatory responses by activating multiple cellular signaling pathways, especially NF-κB and HIF-1α [47, 48]. These pathways not only promote the expression of pro-inflammatory cytokines (such as IL-1β and TNF-α) but also facilitate the formation and activation of osteoclasts at the cellular level, thereby exacerbating the progression of osteoporosis [49]. In osteoarthritis, copper amplifies the inflammatory response by regulating NF-κB and HIF-1α signaling, leading to cartilage degradation and proliferation of synovial cells [32, 50, 51]. This signal amplification effect may lead to excessive proliferation of synovium and formation of rheumatoid nodules, thereby accelerating the damage and deformation of joints [52, 53]. Furthermore, copper, by regulating the signaling pathways in the synovial membrane and the bone marrow cavity, not only amplifies the inflammatory response but also causes the dissolution of the extracellular matrix of bone cells and synovial inflammation, further worsening the degree of inflammation and necrosis in the joint [54]. In the context of tumors, the interaction between copper and signaling pathways enhances the invasiveness of tumors and promotes the inflammatory response in surrounding tissues [55]. In conclusion, copper amplifies inflammatory responses through multiple biological signaling pathways, significantly influencing the process and severity of bone and joint disorders.

## Clinical application of targeted therapeutic strategies for Cuproptosis in bone and cartilage tissues

### Copper chelating agent and copper ion carrier

We have already understood the alterations in copper homeostasis in bone and cartilage, and in combination with the discovery of CRGs, this represents a promising therapeutic target for treating these diseases. The dynamic equilibrium of copper is usually restored through two strategies, namely, using copper chelators to reduce the bioavailability of copper and using copper carriers to transport copper into cells and increase the intracellular copper level. Some common chelating agents for copper include penicillamine, zinc sulfate (ZS), trientine and ammonium tetrasulfotungstate (ATTM) [3] (Table 1). The copper-binding agents are capable of suppressing cell growth by attaching to the surplus copper inside the cells. They can also induce apoptosis of cells through redox reactions to generate a cluster of ROS, selectively target and eliminate senescent chondrocytes, and effectively promote cartilage formation to treat OA. This distinctive benefit sets them apart from other medications and offers great potential for future advancement. There is still much room for the development of new copper chelating agents. Further research in this field is highly anticipated. It is known that copper ion carriers such as escholomar [56], disulfiram (DSF) [57], copper(II) (atsm) [58] and copper(II) (gtsm) [59] analogues can increase cellular copper levels, especially in mitochondria. At present, copper ion carriers have not been widely applied in the treatment of orthopedic diseases. However, elevated copper concentrations have been observed in most cases of bone and joint diseases. The copper ion carrier is capable of enhancing the intracellular copper level and offers distinct benefits when compared to other medications. The discovery and investigation of novel copper ion carriers present significant potential. It is worth expecting that more research will be carried out in this field. Furthermore, endoplasmic reticulum stress can interfere with the homeostasis of non-erythrocytes and trigger the unfolded protein response (UPR). Prolonged or irreversible stress in the endoplasmic reticulum may induce cell death through the activation of the UPR pathway. Therefore, modulating endoplasmic reticulum stress or disrupting the UPR signaling pathway to decrease chondrocyte mortality could represent a potential strategy for the future management of osteoarthritis [60]. Nanoparticles are usually integrated into modern cartilage tissue engineering methods to enhance the functions of chondrocytes, and show promising prospects in the treatment of degenerative joint pathologies [61]. The multifunctional composite thermosensitive hydrogel (HPP@Cu gel) effectively alleviates inflammation by scavenging reactive oxygen species and reactive nitrogen species, regulating macrophage reprogramming, and inhibiting the production of inflammatory mediators. In the OA rat model, these activities have been demonstrated to be capable of alleviating cartilage degeneration and reducing the production of inflammatory factors [62].

**Table 1:** Multiple potential intervention approaches for copper homeostasis.

Therapeutic strategy	Application mechanism	Related research and functions
Copper chelating agent	Reduce the bioavailability of copper and combine with excessive copper to inhibit cell proliferation.	It includes penicillamine, zinc sulfate (ZS), trientine and ammonium tetrathiomolybdate (ATTM), which induce apoptosis through redox reactions, eliminate senescent chondrocytes, promote cartilage formation, and are particularly effective in the treatment of osteoarthritis (OA).
Copper ionophore	Increase intracellular copper levels, especially in mitochondria.	It includes eschmol, disulfiram (DSF), copper (II) (atsm) and copper (II) (gtsm) analogues, increasing copper concentration, and plays a role in most osteoarticular diseases.
Endoplasmic reticulum stress targeting	Interfere with the unfolded protein response (UPR) to reduce cell death.	It targets endoplasmic reticulum stress itself or UPR signals, reduces chondrocyte death, and may become a method for the treatment of OA in the future.
Nanoparticles	Enhance chondrocyte function and reduce inflammation.	It is integrated into cartilage tissue engineering and shows significant therapeutic prospects, significantly reducing cartilage degeneration and the production of inflammatory factors, and improving the condition of OA rat models.
Multifunctional thermosensitive hydrogel	Eliminate reactive oxygen and nitrogen species, regulate macrophages, and inhibit inflammatory mediators.	HPP@Cu gel has been proven to be significantly effective in reducing inflammation in rat models of osteoarthritis, alleviating degeneration and decreasing inflammation.

Although copper chelators (such as penicillamine, zinc sulfate, etc.) and copper ion carriers (such as elesclomol, disulfiram, etc.) have shown potential in regulating copper levels, translating these into orthopedic clinical treatments requires careful consideration of their specificity of action. The key issue lies in balancing therapeutic effects with physiological needs: widely used copper chelators may indiscriminately remove both pathological and physiological copper pools, thereby affecting the normal function of copper-dependent enzymes like lysyl oxidase (LOX); meanwhile, copper ion carriers without targeted delivery may cause collateral damage to healthy bone cells. Therefore, next-generation copper regulation strategies should focus on developing microenvironment-responsive drugs—for example, “smart” chelators or ion carriers that can specifically release active components in inflamed or tumor regions—thus eliminating pathological copper deposits while maximizing protection of physiological copper homeostasis.

#### Therapeutic window: balancing cuproptosis and copper homeostasis

Although strategies targeting cuproptosis (including copper chelators and copper ion carriers) have shown great therapeutic potential in basic research, the core challenge for their successful clinical translation lies in establishing a safe and effective “therapeutic window.” This means precisely distinguishing and intervening in pathological copper overload while strictly preserving physiological copper homeostasis [63]. Systemic administration of copper chelators without selectivity can reduce overall copper levels but easily leads to copper deficiency, impairing the activity of key copper-dependent enzymes such as lysyl oxidase (LOX) and cytochrome c oxidase. Inhibition of LOX activity directly disrupts the cross-linking of collagen fibers and elastin, resulting in decreased bone matrix strength and impaired cartilage elasticity [64]. Dysfunction of cytochrome c oxidase compromises mitochondrial respiratory chain efficiency, affecting the normal function of osteoblasts and chondrocytes, which require high energy supply [65]. Conversely, non-selective use of copper ion carriers may expose normal tissues to high copper environments, triggering unexpected oxidative stress and cell death, leading to off-target toxicity. Therefore, the breakthrough for future therapies lies in achieving precise, selective regulation of cells and lesions, which requires the synergistic implementation of multiple cutting-edge strategies: First, by utilizing intelligent targeted delivery systems—such as the thermosensitive hydrogels or various functionalized nanoparticles proposed in this review—as “smart carriers” for copper-modulating drugs. These systems can actively concentrate the drugs in lesion areas through local injection (e.g., into the joint cavity) or surface-modified targeting ligands (e.g., targeting specific antigens on diseased synovium or osteosarcoma) [66]. Controlled and sustained drug release can be achieved by regulating the degradation rate of the materials or their responsiveness to external stimuli (such as pH or reactive oxygen species levels), thereby maintaining effective therapeutic concentrations locally at the lesion site while minimizing disruption to systemic copper homeostasis and other organs [67]. Second, by exploiting the unique metabolic dependencies of pathological cells—for example, osteosarcoma cells typically exhibit high levels of mitochondrial respiratory activity, which may render them more sensitive to FDX1-mediated cuproptosis. In rheumatoid arthritis, highly proliferative inflammatory synovial fibroblasts and activated immune cells often exist in a state of “metabolic reprogramming,” where their copper uptake mechanisms (such as CTR1) may be upregulated or their antioxidant reserves (such as GSH) relatively insufficient, making them more vulnerable to copper ion-induced oxidative stress [53]. This intrinsic difference in sensitivity creates a valuable “therapeutic index” for copper ion carriers or chelators, enabling the targeted clearance or delivery of copper elements within specific pathological contexts. At the same time, leveraging biomarker-guided precision therapy, by monitoring the expression profiles of cuproptosis-related genes (CRGs) such as FDX1 and LIAS in patient tissues or bodily fluids, as well as measuring free copper levels in serum or synovial fluid as dynamic indicators, we can identify patient subgroups most likely to benefit from copper-targeted treatments (for example, osteosarcoma patients with high FDX1 expression) [68]. Drug dosages can then be adjusted in real time to achieve “on-demand intervention,” ensuring that therapeutic measures are initiated only when copper homeostasis is confirmed to be disrupted in pathological conditions, and discontinued once homeostasis is restored, thereby avoiding the potential risks associated with long-term intervention [69].

### The potential and application prospects of natural substances in the prevention and treatment of oxidative stress induced by cuproptosis

In addition to copper chelators that can suppress copper-induced oxidative stress, research has also demonstrated that certain other plant extracts serve a comparable function. Many chemical substances found in sage, such as rosmarinic acid, silymaric acid and silymarin, all have anti-inflammatory and antioxidant effects (Table 2). These compounds may be helpful in reducing inflammatory responses and mitochondrial oxidative stress responses [70, 71]. Houcine et al. [72] discovered that sage may protect mice from copper-induced liver and kidney damage through the following mechanisms. Firstly, the antioxidant properties of sage reduce the damage caused by reactive oxygen species. Secondly, it inhibits the production of pro-inflammatory factors. Thirdly, it lowers the enzymatic activities related to superoxide dismutase (SOD), catalase (CAT), and acetylcholinesterase (AChE). Vernonia amygdalina is a small shrub native to the tropical regions of Africa. Research has revealed that its ethanol extract can effectively alleviate copper-induced liver and kidney damage through antioxidant mechanisms [73]. Cinnamon, as a member of the Lauraceae family, is a commonly used plant in traditional medicine. It has attracted attention due to its extensive biological activities [70]. The abundant polyphenol content endows cinnamon with the reputation of being a natural antioxidant. It can effectively eliminate reactive oxygen species (ROS) and chelate metals, which may help alleviate the cytotoxicity induced by copper [74]. Apart from its antioxidant activity, cinnamon also possesses various pharmacological effects, including anti-cancer, lowering blood pressure, antibacterial, anti-inflammatory, insecticidal and cholesterol-lowering properties [75–77]. To sum up, although the exact mechanism of action of the aforementioned drugs is still not fully clear, they can effectively inhibit copper-induced oxidative stress and thereby prevent cell damage caused by copper. However, studies have shown that components of traditional Chinese medicine, such as curcumin, can activate the Nrf2/HO-1 pathway, inhibit oxidative stress and cell apoptosis, thereby protecting the body from the cytotoxic effects induced by copper [77, 78]. Furthermore, probiotics are a type of microorganisms that promote the health of the host by regulating the balance of intestinal flora [79]. Previous studies have found that probiotics have the ability to bind and remove heavy metals such as cadmium and lead from the body [80]. A recent study has revealed that probiotics can resist the cytotoxicity induced by copper sulfate by regulating oxidative stress, inflammation and apoptosis [74]. These findings highlight the diverse potential of natural substances in combating oxidative stress induced by copper and other metals. They not only provide new insights into how organisms can be protected in environments rich in metals, but also offer important basis for the development of new therapeutic strategies. Further research should focus on the molecular mechanisms of these natural compounds and optimize their application in the prevention and control of metal toxicity. By integrating modern technology and biomedical research, we can expect to make further progress in terms of safety and efficacy, explore the possibilities of these natural substances in clinical applications, and bring more benefits to individuals affected by metal toxicity.

**Table 2:** The multifunctional properties of various natural extracts and their potential in inhibiting copper-induced oxidative stress and cellular damage.

Natural substances	Main function	Relevant research and mechanisms
Sagebrush	Antioxidant and anti-inflammatory.	It contains rosmarinic acid, silymarin acid and silymarin alcohol, etc., which can reduce the damage of reactive oxygen species, inhibit pro-inflammatory factors, and lower the activities of SOD, CAT and AChE.
Vernonia amygdalina	Antioxidant.	The ethanol extract improves copper-induced cytotoxicity through antioxidant mechanisms.
Cinnamon	Antioxidant, anti-cancer, blood pressure lowering, antibacterial, anti-inflammatory, insecticidal, cholesterol lowering.	High polyphenol content, scavenging reactive oxygen species and chelating metals, reducing copper-induced cytotoxicity.
Curcumin	Antioxidant and anti-apoptotic.	It can activate the Nrf2/HO-1 pathway, inhibit oxidative stress and apoptosis, and protect the body from copper-induced cytotoxicity.
Probiotics	Heavy metal binding, regulating the balance of flora, antioxidation and anti-inflammation.	It can combine and eliminate heavy metals such as cadmium and lead in the body, regulate oxidative stress, inflammation and apoptosis, and resist the cytotoxicity caused by copper sulfate.

### The pathological mechanisms and therapeutic potential of cuproptosis-related genes (CRGs) in orthopedic diseases

Cuproptosis, as a programmed cell death mechanism, plays a significant role in the pathological processes of osteoarthritis (OA), rheumatoid arthritis (RA), osteosarcoma (OS), and osteoporosis (OP). CRGs (Copper Death-related Genes) have demonstrated unique biological functions and clinical relevance in these diseases (Table 3). In OA, the expressions of CRGs such as FDX1, LIPT1, PDHA1, PDHB, DLAT and CDKN2A are significantly elevated in the synovium, which are closely related to synovial inflammation and cell apoptosis. Meanwhile, the high expression of GLS is associated with enhanced immune response and plays an important role in the pathogenesis of OA synovitis [32, 33, 81]. In RA patients, abnormal expressions of CRGs within synovial fibroblasts, such as DLST, DLD and ATP7A, are closely related to the regulation of glycolysis and inflammatory signals. The abnormal expression of FDX1 triggers inflammatory responses in immune cells by influencing fatty acid oxidation and steroid conditions [26, 53, 82, 83]. In OS, abnormal expressions of CRGs such as DLAT, FDX1 and CDKN2A have a significant impact on the tumor immune microenvironment and treatment response. Especially in the context where high expression of FDX1 promotes tumor malignancy progression, it is demonstrated that CRGs play a crucial regulatory role in OS [84–89]. Furthermore, in osteoporosis, PDHA1 and PDHB, as important components of the pyruvate dehydrogenase complex, regulate the energy metabolism of osteoblasts through glycolysis and the TCA cycle [90, 91]. GLS affects the process of cell differentiation by influencing the metabolism of glutamine [29], LIAS enhances mitochondrial function and antioxidant activity through the synthesis of lipoic acid [92], DLAT is involved in energy metabolism regulation [93], CDKN2A is associated with cellular senescence and inflammatory responses [94, 95]. DLD and LIPT1 also play key roles in maintaining energy metabolism and cellular homeostasis [96–99]. The significant biological functions of these genes in various skeletal diseases have revealed their broad potential as diagnostic markers and therapeutic targets. Through the functional analysis of CRGs, on the one hand, it deepens our understanding of the mechanisms of these complex diseases; on the other hand, it provides theoretical support and research basis for future personalized treatment.

**Table 3:** This summary table demonstrates the systemic role of CRGs as potential therapeutic targets in orthopedic diseases and their broad application prospects.

Type of disease	Key CRGs and their mechanisms of action	Therapeutic potential and targets
Osteoarthritis (OA)	High expression of FDX1, LIPT1, PDHA1, PDHB, DLAT, and CDKN2A, and high expression of GLS, enhances immune response.	Therapeutic targets focus on synovial inflammation and apoptosis, possibly alleviating symptoms by inhibiting inflammatory pathways.
Rheumatoid arthritis (RA)	Abnormal expressions of DLST, DLD and ATP7A affect glycolysis and inflammatory signals, while abnormal expression of FDX1 influences inflammatory responses of immune cells.	By regulating fatty acid oxidation and steroid synthesis, targeted intervention in the abnormal responses of immune cells can alleviate related inflammatory symptoms.
Osteosarcoma (OS)	Abnormal expression of DLAT, FDX1, and CDKN2A affects the tumor microenvironment and treatment response. High expression of FDX1 promotes the malignant progression of tumors.	By regulating the tumor immune microenvironment to restrict tumor cell proliferation or invasion, it provides a new perspective for disease management.
Osteoporosis (OP)	PDHA1 and PDHB regulate energy metabolism in osteoblasts, GLS affects cell differentiation, LIAS enhances mitochondrial function and antioxidant capacity, DLAT regulates energy metabolism, and CDKN2A is associated with aging and inflammation.	Promoting osteoblast metabolism and intervening in the pathways related to aging and oxidative stress are expected to improve the symptoms of osteoporosis.

### The key roles of exosomal miRNAs in cuproptosis and cell regulation, as well as potential therapeutic strategies

Exosomal miRNAs play a crucial regulatory role in copper homeostasis. They exert significant biological effects by regulating multiple genes (Table 4). Among them, the imbalance of copper homeostasis is closely related to several regulatory factors. The negative regulators consist of glutaminase (GLS), cyclin-dependent kinase inhibitor 2A (CDKN2A), and metal-regulated transcription factor 1 (MTF1). These typically suppress the biological activities that are exaggerated by harmful substances. Meanwhile, the relevant positive regulatory factors include ferredoxin 1 (FDX1) and various acylated proteins, specifically including lipid acyltransferase 1 (LIPT1), lipoic acid synthase (LIAS) and dihydrolipoamide dehydrogenase (DLD). These proteins are involved in the lipoic acid synthesis pathway and play an important role in maintaining cellular metabolic functions [4].

**Table 4:** The key roles of exosomal miRNAs in cuproptosis and cell regulation, as well as potential therapeutic strategies.

Exosomal miRNA/factors	Main functions and mechanism of action	Potential therapeutic strategies and their impacts
miR-21-5p	Targeting PDHA1 to enhance glycolysis and reduce the sensitivity to chemotherapy drugs.	It can be used to regulate glycolysis, influence cellular metabolism, and potentially be applied to the regulation of cancer cell sensitivity.
miR-4536-5p	Inhibition of PDHB expression affects osteoclast differentiation.	By regulating the osteoclast pathway, it may affect the treatment of bone metabolism-related diseases.
miR-663a	Regulate CDKN2A and repair hypoxia-induced damage.	By promoting cellular stress recovery, it is applied to the repair of hypoxia-related conditions.
miR-182	Targeting TLR4 promotes the transformation of macrophages to the M2 phenotype and reduces inflammatory responses.	Regulating the inflammatory response pathway helps reduce inflammation-related damage and is applied in immunomodulatory therapy.
Glutaminase (GLS)	Maintaining copper homeostasis.	As a negative regulatory factor, it may be used for the regulation of copper homeostasis and pathological intervention.
Ferredoxin 1 (FDX1)	Influencing metabolic function through the lipoic acid synthesis pathway.	As a positive regulatory factor, it supports copper-related metabolic pathways and is applied in metabolic-related interventions.
The TLR4/NF-κB and MAPK signaling pathways	Regulating miRNAs to reduce inflammation, oxidative damage and apoptosis, and prevent copper-related cytotoxicity.	Exploring the intervention of signal pathways may be a therapeutic strategy for various inflammatory and related tumor diseases.

Studies have shown that exosomal miR-21-5p extracted from cisplatin-resistant SKOV3 ovarian cancer cells enhances glycolysis by targeting PDHA1 and reduces the chemosensitivity of cells to the drug [100]. PDHA1, a gene associated with copper poisoning, has an important function in glucose metabolism, the TCA cycle, and mitochondrial oxidative phosphorylation [101]. The suppression of PDHA1 might enhance the proliferation of osteoblasts, whereas its stimulation could influence the functions of macrophages and osteoblasts by reducing the secretion of inflammatory factors [31].

Furthermore, the exosomes of fibroblasts from keloid scars of human beings can inhibit the expression of PDHB. And PDHB may affect osteoclast differentiation through interacting with NIMA-related kinase 10 [31]. The miR-663a released by exosomes of human umbilical cord mesenchymal stem cells (hucMSCs) can repair the damage of hypoxia-induced endometrial epithelial cells by regulating CDKN2A [102]. The miR-182 derived from bone marrow mesenchymal stem cells alters the polarization state of macrophages by targeting TLR4, promoting their transformation into the anti-inflammatory M2 phenotype and reducing the overall inflammatory response [103].

By regulating the TLR4/NF-κB and MAPK signaling pathways, exosomal miRNAs can effectively reduce inflammatory responses, oxidative damage and apoptosis, thereby preventing copper-related cytotoxicity [104]. Investigating the miRNAs in vesicles and their interactions with copper homeostasis provides us with a new perspective to understand the complex molecular mechanisms underlying them in biology. Through these studies, understanding these gene regulatory networks may provide important intervention strategies for addressing copper-related pathologies.

### An innovative approach to improving the treatment of orthopedic diseases by multi-dimensional regulation of Cuproptosis through a hydrogel sustained-release strategy

Based on the existing research, we have discovered that cuproptosis is not a single form of programmed cell death, but rather a multi-dimensional cell death mechanism that is intricately associated with mitochondrial oxidative stress and complex intercellular signaling. This complex pathological feature significantly increases the challenge of treating orthopedic-related diseases by targeting cuproptosis. For instance, excessive copper ions may trigger mitochondrial oxidative stress responses within cells and simultaneously disrupt normal cellular functions through the cell communication network. This not only involves pathological changes within individual cells, but also further extends to pathological evolution at the levels of tissues and systems.

**Fig. 2:**
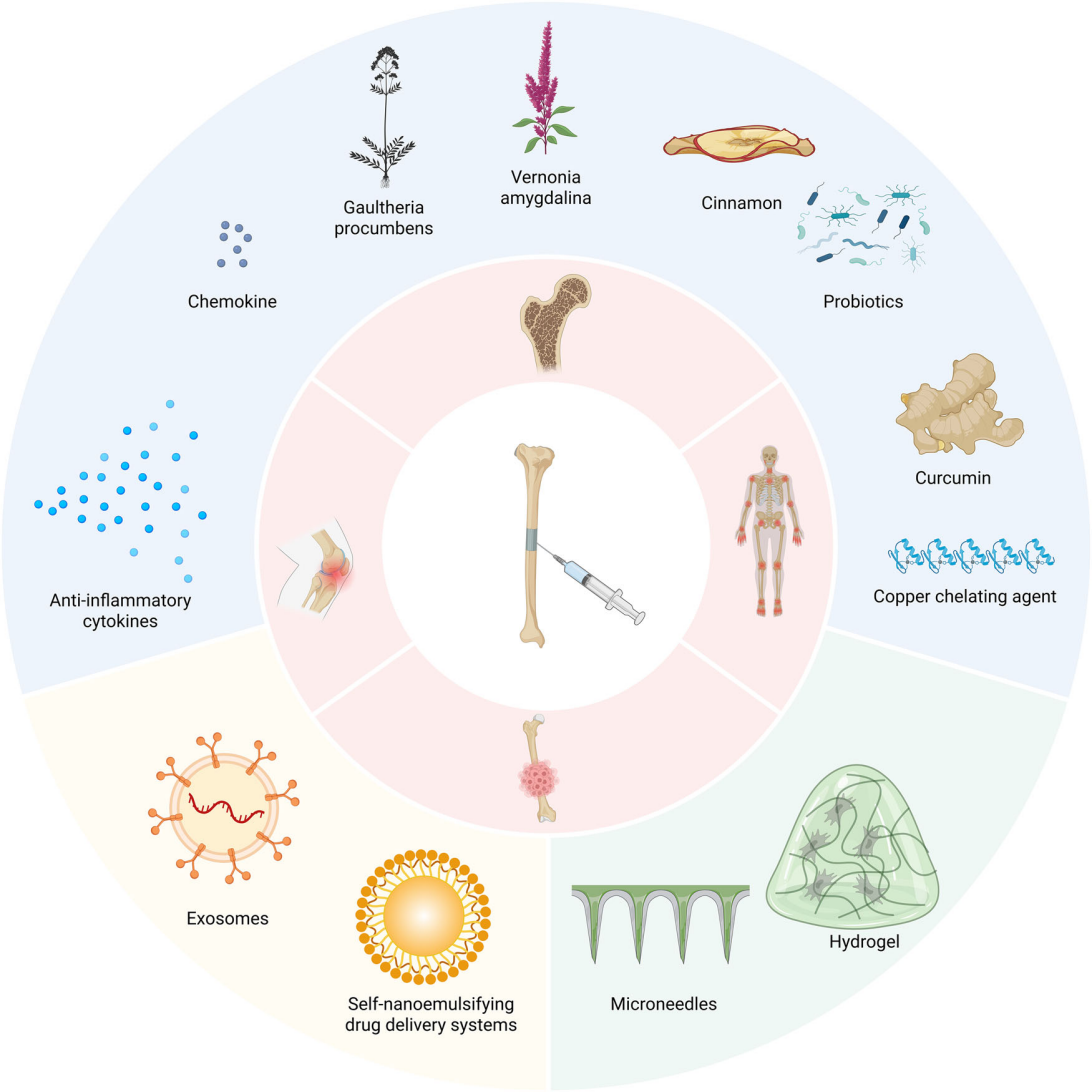
An innovative approach to improving the treatment of orthopedic diseases by multi-dimensional regulation of Cuproptosis through a hydrogel sustained-release strategy.

In the face of these challenges, our team has proposed an innovative solution. We hypothesize that by using hydrogels as carriers to load known natural drugs and by controlling the crosslinking density of the hydrogels to achieve sustained drug release, it is possible to multi-dimensionally target and inhibit cuproptosis (Fig. 2). The physical properties of hydrogels, such as flexibility and porosity, enable us to precisely regulate the drug release time and rate according to therapeutic requirements. This sustained-release mechanism not only reduces the side effects of the drug on the internal organs when it passes through the gastrointestinal tract, but also ensures that the drug is more accurately concentrated at the lesion site, thereby enhancing the targeted and effective nature of the treatment.

Furthermore, the biocompatibility and degradation characteristics of hydrogels enable long-term implantation in the body and their natural degradation after the completion of the treatment, without imposing a persistent burden on the internal environment. We envision that this innovative approach can precisely regulate the cuproptosis process by maintaining an ideal drug concentration in the lesion area, thereby not only significantly enhancing the therapeutic effect but also reducing the overall treatment risk.

We plan to conduct a series of systematic research and experiments, including in vitro model verification, animal experiments, and preliminary clinical trials. Through these experiments, not only will the clinical feasibility and effectiveness of this strategy be verified, but also the combination of the improved hydrogel with various natural drugs will be explored. Through customized treatment plans, individual differences will be addressed. We are committed to providing safer and more effective treatment options for patients suffering from orthopedic diseases, and promoting the application prospects of personalized medicine in orthopedic pathological treatment. Our long-term goal is to expand this technology to a broader range of disease fields and bring innovative solutions to the related medical challenges.

## Summary and outlook

Cuproptosis, as a novel programmed cell death mechanism, has demonstrated a crucial role in various orthopedic diseases such as osteoporosis, osteoarthritis, rheumatoid arthritis and osteosarcoma. It induces oxidative stress, damages mitochondrial function and activates inflammatory responses, thereby forming a complex pathological pathway that poses a threat to the health of bone and cartilage tissues. Studies have shown that the expression of copper homeostasis imbalance-related genes (CRGs) and cuproptosis in these diseases provides important diagnostic markers and therapeutic targets. To address the challenges brought about by cuproptosis, we need to explore new therapeutic strategies. This prospect encompasses the application of copper chelators, copper ion carriers, as well as natural substances and exosomal miRNAs. These strategies aim to regulate copper levels and intervene in the pathological processes triggered by copper through multi-dimensional intervention. The drug sustained-release system based on hydrogel has demonstrated the potential to enhance the efficiency and targeting of drug delivery, and to precisely and efficiently control the process of cuproptosis.

In the future, a thorough understanding of CRGs and their mechanisms and roles in cuproptosis will be the focus of research. This will not only promote the development of precision medicine but also guide the design and implementation of personalized treatment plans. Through interdisciplinary collaboration and the impetus of innovative technologies, the effective management of cuproptosis will not only improve the quality of life of patients, but also provide a blueprint for other pathological studies related to metal ions. The future of cuproptosis therapy lies not in the blanket depletion of copper, but in the sophisticated, context-dependent restoration of copper homeostasis. Further basic and clinical research will undoubtedly deepen our understanding of copper and its role in orthopedic diseases, laying a solid foundation for achieving safer and more efficient treatment plans.

Looking ahead, research on CRGs should go beyond merely describing expression profiles and focus on addressing several core challenges: First, it is essential to elucidate the “cross-talk” mechanisms of CRG networks among different cell types within the skeletal system, particularly the regulatory role of CRGs in osteoclast-derived exosomes on osteoblast fate. Second, leveraging single-cell multi-omics technologies and gene editing tools to establish causal regulatory maps of CRGs in orthopedic diseases is crucial. Most importantly, efforts should be made to advance CRGs from basic research toward clinical translation by exploring their potential as actionable therapeutic targets—for example, developing small-molecule drugs that specifically modulate CRG activity in local bone tissue while avoiding systemic disruption of copper homeostasis.
